# Carm1 and the Epigenetic Control of Stem Cell Function

**DOI:** 10.1093/stcltm/szac068

**Published:** 2022-09-14

**Authors:** John Saber, Michael A Rudnicki

**Affiliations:** Sprott Centre for Stem Cell Research, Regenerative Medicine Program, Ottawa Hospital Research Institute, Ottawa, ON, Canada; Department of Cellular and Molecular Medicine, Faculty of Medicine, University of Ottawa, Ottawa, ON, Canada; Sprott Centre for Stem Cell Research, Regenerative Medicine Program, Ottawa Hospital Research Institute, Ottawa, ON, Canada; Department of Cellular and Molecular Medicine, Faculty of Medicine, University of Ottawa, Ottawa, ON, Canada

**Keywords:** Carm1, asymmetric division, autophagy, pre-mRNA splicing, muscle stem cells

## Abstract

Coactivator-associated arginine methyltransferase 1 (CARM1) is a methyltransferase whose function has been highly studied in the context of nuclear receptor signaling. However, CARM1 is known to epigenetically regulate expression of several myogenic genes involved in differentiation such as *Myog* and *MEF2C*. CARM1 also acts to regulate myogenesis through its influence on various cellular processes from embryonic to adult myogenesis. First, CARM1 has a crucial role in establishing polarity-regulated gene expression during an asymmetric satellite cell division by methylating PAX7, leading to the expression of *Myf5*. Second, satellite cells express the CARM1-FL and CARM1-ΔE15 isoforms. The former has been shown to promote pre-mRNA splicing through its interaction with CA150 and U1C, leading to their methylation and increased activity, while the latter displays a reduction in both metrics, thus, modulating alternative pre-mRNA splice forms in muscle cells. Third, CARM1 is a regulator of autophagy through its positive reinforcement of AMPK activity and gene expression. Autophagy already has known implications in ageing and disease, and CARM1 could follow suite. Thus, CARM1 is a central regulator of several important processes impacting muscle stem cell function and myogenesis.

## Graphical Abstract

**Graphical Abstract:**
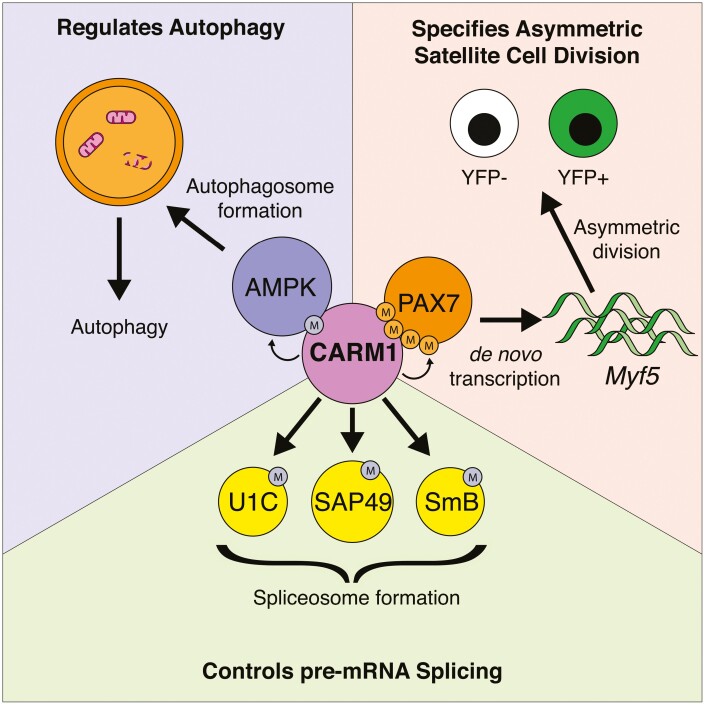
CARM1 is involved in numerous aspects of cellular function. CARM1 can specify satellite cell asymmetric divisions by regulating the expression of *Myf5*. CARM1 can regulate pre-mRNA splicing by methylating various components of the spliceosome. CARM1 can regulate autophagy by methylating AMPK, supporting the formation of autophagosomes.

## Introduction

Epigenetics is the modulation of gene expression without modifying the underlying DNA sequences. This can be done in multiple ways, such as DNA methylation, chromatin organization, and histone modifications. A combination of all 3 are involved in the temporal regulation of gene expression throughout development and are responsible for generating different cell types. In muscle stem cells, also known as satellite cells, histone modifications are the most studied subsets of epigenetics. In this regard, multiple types of histone modifications have been identified to play a role in their function. H3K4me3 and H3K27me3, activating and repressing histone modifications respectively, can simultaneously occupy gene promoters in a bivalent state.^1^ Additionally, the presence of only one is associated with its respective function. In quiescent satellite cells in skeletal muscle, the majority of promoters are marked by H3K4me3 and are devoid of H3K27me3. However, during activation, many promoters are quick to acquire the repressive mark indicating that large-scale repression of gene expression is necessary to exit quiescence.^1^

CARM1, also known as PRMT4, is a transcriptional coactivator that associates itself with a variety of transcription factors to asymmetrically dimethylate them or chromatin and is thus involved in the epigenetic regulation of gene expression.^2,3^ CARM1 asymmetrically dimethylates Histone 3 at arginine 17 (H3R17me2a), and this modification is associated with transcriptional activation.^4^

The epigenetic function of CARM1 was first described in the context of the control of nuclear receptors through methylation of the p160 family of coactivators and p300/CPB.^5,6^ This in turn leads to histone acetylation, and subsequent gene activation. Moreover, CARM1 has been shown to interact with members of the SWI/SNF family of chromatin remodelers to induce estrogen signaling.^4^ Simultaneously, methylation of histone by CARM1 leads to a reduction in binding of the nucleosome remodeling and deacetylase complex (NuRD), which functions to maintain histone acetylation and active gene transcription.^7^

Although much is known about CARM1’s general ability to regulate gene transcription through its modifications to histones and transcription factor coactivators, its methyltransferase activity is also involved in a variety of cellular processes. In this concise review, we will examine CARM1’s multiple roles in the regulation of myogenesis, in the regulation of pre-mRNA processing, and in the regulation of autophagy.

## Role of CARM1 in Myogenesis

Satellite cells, also known as muscle stem cells, are cells residing between the basal lamina and the lipid membrane of the muscle fiber.^8^ These cells are characterized by the expression of the transcription factor PAX7.^9-11^ Under homeostatic conditions, satellite cells are in a quiescent state.^12^ However, following injury, these cells become activated, proliferate, and eventually differentiate to replace damaged muscle fibers. All of these steps are marked by the sequential expression of various myogenic regulatory factors such as MYF5, MYOD, MYOG, and MYF6.^13^ In general, arginine methylation is important for the proliferation of cells and in the maintenance of stem cells. In addition, it has been suggested that CARM1 acts to counter replicative senescence in fibroblasts.^14^ Therefore, there is interest in understanding the role of CARM1 in stem cell populations such as satellite cells.

### CARM1 Controls Cell Differentiation

Myogenic cell differentiation is regulated by the successive expression of MYOG, MEF2C, and MYF6.^13^ CARM1 has been identified as an important factor governing terminal muscle differentiation by binding to MEF2C to modulate target gene expression, such as creatine kinase.^15^ CARM1 also has the possibility to play a role in early muscle differentiation by activating *Myog* expression, although this was identified in the context of rhabdomyosarcoma-derived cells.^16^

Arginine methylation appears to be crucial for proper muscle differentiation. PRMT5 has been shown to be required for early skeletal muscle differentiation by associating to the *Myog* promoter causing histone demethylation and facilitating ATP-dependent chromatin remodeling through mediating BRG1 binding.^17^ In that same light, PRMT5 and CARM1 are involved in the later stages of differentiation by also binding to, methylating, and recruiting BRG1 and the rest of the SWI/SNF complex of chromatin remodelers at late genes, such as *Ckm* and *Des*.^18^ Interestingly, this was lost in the absence of CARM1, indicating that although PRMT5 is also able to bind late genes, only CARM1 is absolutely required for their remodeling.

Lastly, knockdown of *Carm1* in C2C12 myoblasts suggested a role for regulating glycogen metabolism during differentiation.^19^ siRNA knockdown of *Carm1* resulted in decreased expression of *Gys1*, *Pgam2*, and *Pygm*, all of which are genes involved in glycogen metabolism. Furthermore, glycogen storage was impaired in a methylation mutant of CARM1, indicating that CARM1 has an important role in glycogen metabolism in muscle.

### CARM1 Is a Mediator of Satellite Cell Fate Decision

During the activation phase, satellite cells make a choice to either divide symmetrically or asymmetrically.^20^ The different modes of divisions are detected using the *Myf5-Cre/ROSA26-YFP* mouse model, whereby cells that undergo *de novo* transcription of *Myf5* are permanently labeled with the YFP reporter.^20^ In a symmetric division, satellite cells divide to generate 2 identical daughter cells that either express *Myf5* (becoming activated) or do not (return to quiescence). However, in an asymmetric division, the activated daughter cell expresses *Myf5*, whereas the muscle stem cell does not, ensuring that both the demand for regeneration and homeostasis can be met. CARM1 plays a pivotal role in that decision-making process and is thus an important mediator in satellite cell fate.

During an asymmetric division, the satellite cell becomes polarized along the axis of division based on the expression of DMD (dystrophin), MARK2, and PARD3.^21^ This polarization of DMD (and by extension, the dystrophin glycoprotein complex of which DMD is a part of) to one side of the cell creates a microenvironment favorable for the initiation of an asymmetric cell division.

**Figure 1.**
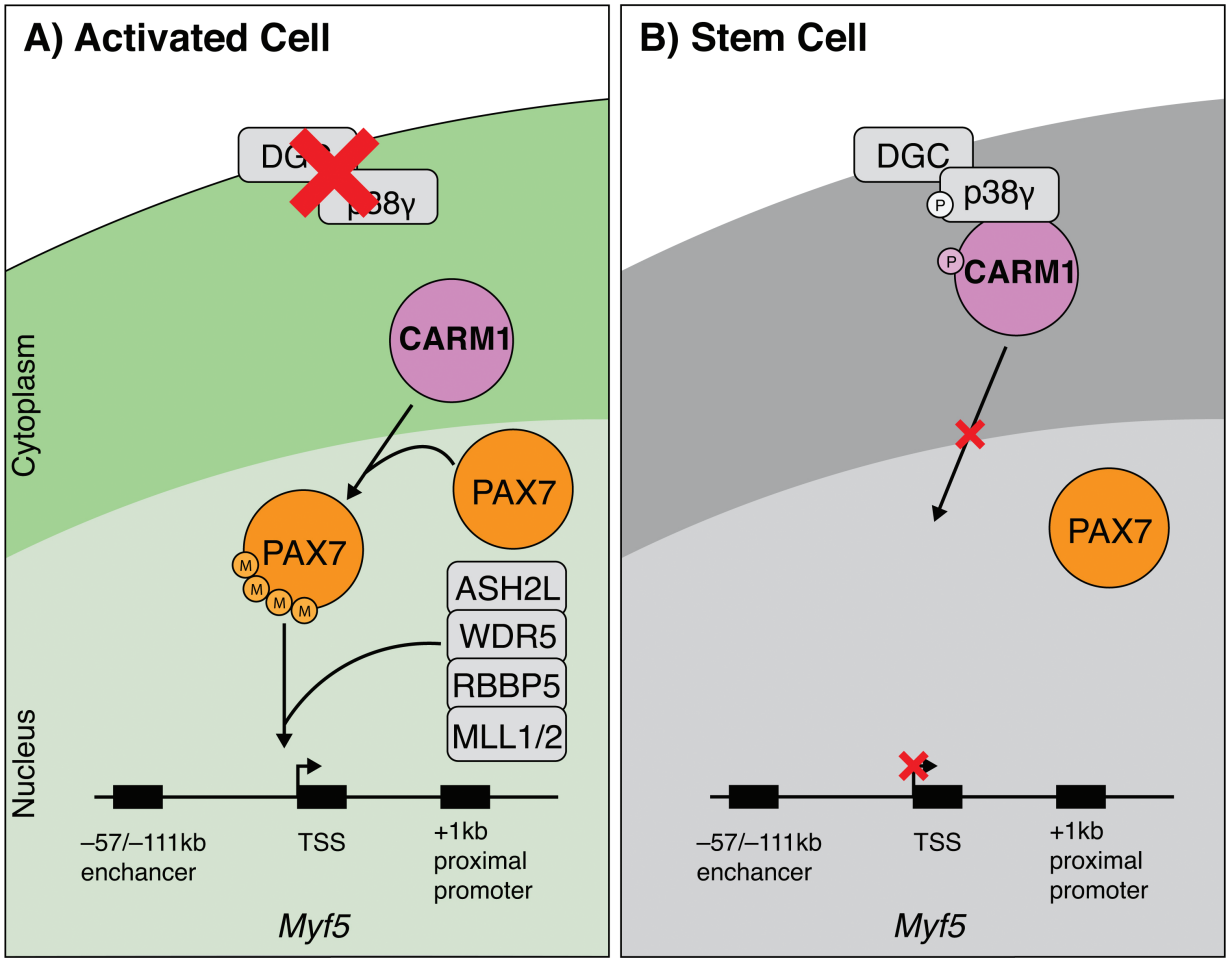
Schematic representation of a CARM1-mediated asymmetric satellite cell division. The dystrophin glycoprotein complex (DGC) polarizes the cell, leading to divergent fates of the daughter cells (**A**) In the satellite cell undergoing activation, the dystrophin glycoprotein complex (DGC) is missing. This allows CARM1 to freely enter the nucleus, where is methylates PAX7. Methylated PAX7 then recruits the histone methyltransferase complex comprising of MLL1/2, ASH2L, WDR5, and RBBP5, leading to the induction of *Myf5* transcription. **(B)** In the satellite stem cell, the presence of dystrophin leads to its interaction with p38γ. This results in heightened phosphorylation of p38γ and CARM1, leading to its sequestration to the cytoplasm, resulting in a reduction in *Myf5* expression.

On the side where DMD is absent, facing the muscle fiber, CARM1 enters the nucleus normally and methylates PAX7.^22,23^ Methylated PAX7 can then associate with the histone methyltransferase complex containing MLL1/2, WDR5, ASH2L, and RBBP5, allowing the expression of PAX7 target genes, namely *Myf5* in this case (Fig. 1A).^23,24^ This cell becomes committed to becoming a progenitor and thus contributes to muscle regeneration. By contrast, on the DMD-expressing side facing the basal lamina, CARM1 binds to and is phosphorylated by p38γ at the dystrophin glycoprotein complex.^22^ This blocks CARM1 from translocating to the nucleus preventing the methylation of PAX7 and impairing the expression of *Myf5* (Fig. 1B). This cell then returns to quiescence maintaining the stem cell pool.

Furthermore, CARM1 can contribute to satellite cell asymmetric divisions by its actions on Notch signaling. NOTCH1 is known to be expressed on the satellite cell membrane and interacts with its ligand DLL1.^25^ Notch signaling is well-known for its ability to maintain satellite cells in a quiescent state.^25-27^ The interaction between NOTCH1 and DLL1 leads to the cleavage of the notch intracellular domain (NICD) which translocates to the nucleus, binds to the transcription factor RBPJ, and can mediate gene transcription of target genes, such as *Hes* and *Hey* genes. Interestingly, during an asymmetric division, the daughter cells also have an asymmetric localisation of DLL1 seemingly linked to *Myf5* expression.^20^ This suggests that the cell undergoing commitment is interacting with the adjacent cell to maintain stemness, ensuring only one of both cells commit to the myogenic lineage. Moreover, CARM1 has been shown to methylate the NICD at 5 arginine residues, thereby increasing its activity at inducing gene expression.^28^ Mutating these arginine residues led to increased protein stability, but also a drastic decrease in their biological activity.

Corroborating these results, selective ablation of *Carm1* in satellite cells leads to a reduction in asymmetric satellite cell divisions resulting in hyperplasia of satellite cells that do not commit to myogenesis and impaired muscle regeneration.^23^ Perhaps coincidentally, muscles from patients with Duchenne muscular dystrophy and a dystrophic mouse model (*mdx* mouse) also display satellite cell hyperplasia and impaired regeneration.^21,29^ Moreover, satellite cells from *mdx* mice display a significant reduction in asymmetric divisions,^21^ suggesting CARM1 might be misregulated in *mdx*. In fact, the loss of dystrophin leads to the collapse of the dystrophin glycoprotein complex preventing the sequestration of p38γ and CARM1. This results in a heightened phosphorylation of CARM1 throughout the cell and an impairment in PAX7 methylation and *Myf5* expression resulting in reduced asymmetric divisions.^22^ Moreover, the sequestration of CARM1 to the cytoplasm may reduce the effectiveness of Notch signaling since CARM1 would not be able to methylate NICD in the nucleus.

### CARM1 Is a Regulator of Muscle Development

Given the important role that CARM1 plays in adult myogenesis, and the striking similarities between the latter and embryonic myogenesis, it is possible that a similar function might be observed during embryonic myogenesis.^22,23^ CARM1’s earliest known involvement is during the inner cell mass formation of the blastocyst, where it is crucial for maintaining pluripotency and preventing precocious differentiation.^30^ However, it does not seem to maintain the same function in muscle, further reinforcing the previously mentioned notion that CARM1 has cell-specific roles.

Microarray analysis of the myotome of trout embryos suggests elevated expression of a number of genes involved in transcriptional regulation including *carm1*.^31^ During muscle development in zebrafish, Carm1 was shown to be involved in the regulation of *myog* expression, as well as the formation of fast twitch myofibers.^32^ Knockdown of *carm1* in zebrafish using antisense morpholinos led to smaller somites, reduced *myog* and *mef2c* expression, and a loss of *mlc2f* expression leading to a reduction in fast fiber formation. These data further suggest a role for CARM1 in early myogenesis, and the induction of differentiation. However, the role of CARM1 in muscle embryonic development in mice has not been well studied.

CARM1 plays significant roles at all levels of myogenesis. It is involved in regulating embryonic myogenic differentiation, as well as adult muscle differentiation. More importantly, CARM1 plays a crucial role as a master regulator of cell fate during satellite cell asymmetric divisions.

## CARM1 Is a Regulator of mRNA Processing

RNA processing constitutes all the events from the newly transcribed immature pre-RNA to the mature mRNA that is used for translation. One crucial step is the removal of introns from the pre-mRNA, called splicing, that results in the uninterrupted chain of exons being formed.^33^ Briefly, U1 and U2 proteins recognize the 5ʹ and 3ʹ splice sites, respectively. This allows the recruitment of U4/5, U6, and other proteins such as SAP49 and SmB to form the complete spliceosome which mediates splicing.

### CARM1 Promotes Exon Skipping and Alternative Splicing

**Figure 2.**
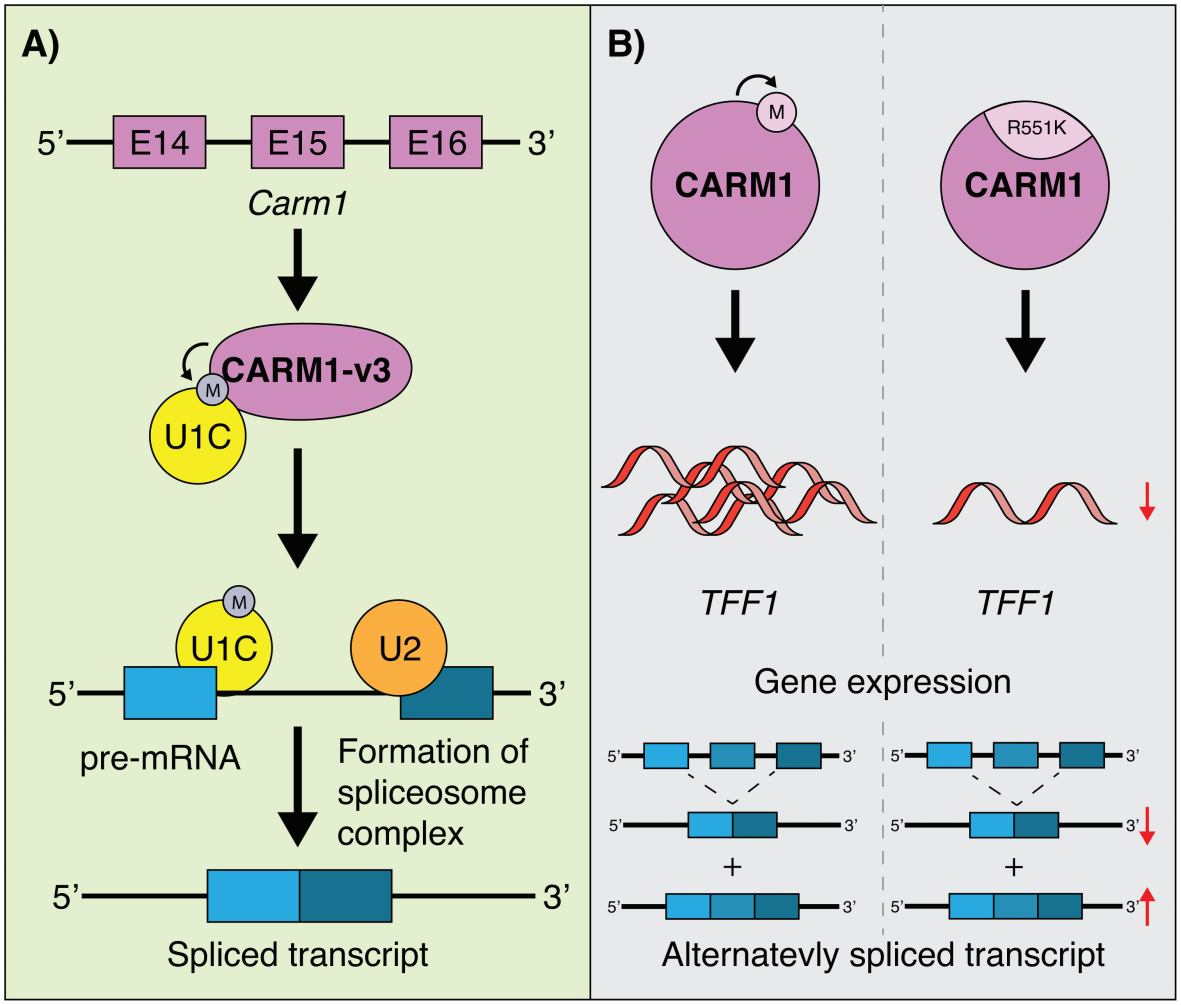
CARM1 regulation of pre-mRNA splicing. (**A**) Retention of introns 15 and 15 of *Carm1* leads to the generation of a different form, called CARM1-v3. CARM1-v3 can interact with and methylate U1C, a member of the spliceosome complex. This favorably affects splicing of pre-mRNA. Moreover, CARM1 can methylate SmB and SAP49, other members of the spliceosome. (**B**) CARM1 contains an automethylation site on arginine 551. Mutating it to a lysine (R551K; methylation deficient) leads to a reduction in its ability to activate gene transcription of ERα target genes (*TFF1* is shown as an example) or promote alternative splicing.

While studies have shown CARM1 can bind to transcriptional machinery and chromatin remodelers, CARM1 has also displayed interactions with RNA-binding proteins such as HuD, which is involved in neuronal differentiation.^34^ This type of interaction introduces the possibility of CARM1 being involved in the regulation of differential splicing of pre-mRNA. In fact, one such study identified that U1C, a member of the U1 snRNP complex, associates with an isoform of CARM1 (CARM1-v3; generated by the retention of introns 14 and 15) to modulate distal 5ʹ splicing of E1A pre-mRNA (Fig. 2A).^35^ CARM1-v1, representing the full-length version of CARM1, does not display this splicing regulation. To determine if CARM1’s methyltransferase activity was important in splicing, CARM1-WT and enzymatically null CARM1 (CARM1-dead) were transfected into CARM1-KO cells and a CD44v5 minigene as a reporter driven by an estrogen response element was used as a readout for alternative splicing. They found that there was a higher incidence of alternatively spliced transcripts in the CARM1-dead condition compared with CARM1-WT, suggesting that CARM1 methyltransferase activity promotes exon skipping. Subsequent studies identified splicing and transcription elongation factors CA150, SAP49, SmB, and U1C as proteins that are specifically methylated by CARM1, further supporting this notion.^3^

### *Carm1* Automethylation Is Important for Its Pre-mRNA Processing Function

CARM1 is expressed in different forms obtained by alternative splicing and whose functions have been studied in the context of pre-mRNA splicing. To control pre-mRNA splicing, CARM1 can be subjected to post-translational modifications. In fact, CARM1 possesses an automethylation site at arginine 551 (R551) on Exon 15.^36^ This methylation does not affect the methyltransferase activity of CARM1. However, CARM1 with a mutated methylation site displayed an inability to activate gene transcription, such as known ERα target genes *TFF1*, *IGFBP4*, and *PTGES*, suggesting that CARM1 automethylation plays indirect roles in gene expression (Fig. 2B).

Interestingly, CARM1 automethylation is also strongly implicated in alternative splicing. A splicing assay between CARM1-WT, CARM1-R551K (automethylation deficient mutation), and a vector containing a CT/CGRP reporter gene lead to a decreased level of CGRP relative to CT, indicating a reduction in splicing of the reporter. Lastly, CARM1 can itself be alternatively spliced into 2 main forms: CARM1-FL (full length), and CARM1-ΔE15 (a version lacking exon 15). Both isoforms retain methyltransferase activity, but CARM1-ΔE15 is missing the automethylation site on exon 15.^37^ Like CARM1-R551K, CARM1-ΔE15 displays a reduction in its coactivator function in mediating gene expression. Although not mentioned in the manuscript, CARM1-ΔE15 may also be regulating alternative splicing similarly to CARM1-R551K. Therefore, *Carm1* alternative splicing seems to be a mechanism used by the cell to control the level of CARM1 methylation. Furthermore, both forms display tissue-specific levels of expression, and it is likely that cells are using *Carm1* alternative splicing to control the level of CARM1 methylation.

These data suggest that different forms of CARM1 retain methyltransferase activity, but that differential regulation of CARM1 can influence specific gene expression and alternative splicing. Given different substrate specificity between different forms of CARM1, it is also possible that another layer of regulation exists when it comes to methylating proteins involved in alternative splicing, such as U1C and CA150.

### Implications of CARM1 Function on Pre-mRNA Splicing in Satellite Cells

Although the regulation of pre-mRNA splicing by CARM1 has not been studied in the context of muscle, some important clues can be obtained from our current knowledge. CARM1 can regulate pre-mRNA splicing based on its own splicing pattern. In muscle, both CARM1-FL and CARM1-ΔE15 are expressed, but only CARM1-ΔE15 can methylate PAX7.^22^ This contrasts with previous findings indicating CARM1-FL can recognize more substrates, implying some level of cell specificity.^37^ This introduces the potential for alternative splicing to occur. Moreover, distinct CARM1 regulation during an asymmetric division may lead to differential pre-mRNA splicing between the activated satellite cell and the one returning to quiescence. The cell expressing *Myf5* would have both forms of CARM1 translocate to the nucleus, where transcriptional regulation and alternative pre-mRNA splicing may occur. However, in the cell not expressing *Myf5*, CARM1 sequestration to the cytoplasm has already known transcriptional consequences, with yet undiscovered splicing implications. Furthermore, given the extent to which CARM1 is misregulated in *mdx* satellite cells, it is also likely that pre-mRNA alternative splicing is misregulated and has a role in disease progression and the regenerative deficit observed in Duchenne muscular dystrophy.

The importance of alternative splicing in the function of stem cells is not a new concept. It has been shown extensively in the context of embryonic stem cells and induced pluripotent stem cells that alternative splicing is tightly associated their ability to maintain stemness and control differentiation.^38^ Moreover, alternative pre-mRNA splicing has been implicated in the specification of the hematopoietic lineage and development^39,40^ and neurogenesis and brain development.^41^ Therefore, a role for alternative splicing in the control of satellite cell activation is consistent with its observed function in other tissue systems.

In summary, CARM1 has been implicated in multiple levels of RNA processing. CARM1 promotes exon skipping in pre-mRNA through its interaction with U1C. Moreover, CARM1 can modulate pre-mRNA splicing by interacting with various spliceosome components such as CA150. Finally, CARM1 itself is subject to differential splicing in order to regulate its ability to process pre-mRNA. Finally, recent research has revealed the potential of CARM1 in regulating mRNA decay through its association with UPF1 in the context of spinal muscular atrophy.^42^ Interestingly, this mechanism applied to engineered mRNAs, and was observed in naturally occurring mRNA, indicating some important biological function.

## CARM1 Is a Regulator of Autophagy

In addition to Duchenne muscular dystrophy, CARM1 is potentially involved in muscle ageing through its contributions to autophagy. Sarcopenia is the age-associated loss of muscle mass and strength. A major contributor to the progression of sarcopenia in geriatric patients is the inability of damaged muscle to undergo proper regeneration.^43,44^ The cause of this regenerative deficit has been suggested to be a consequence of an inability of sarcopenic satellite cells to maintain quiescence and their transition to senescence.^45^ Upon injury, these cells are not able to enter the cell cycle and participate in the normal regenerative process. Moreover, this senescent state appears to be irreversible since transplantation of geriatric satellite cells into a young recipient muscle does not correct the regenerative deficit. Therefore, an imbalance during the ageing process of satellite cells has long-lasting effects.

Dysregulation of autophagy has been suggested to play a role in the gerontological changes that occur in satellite cells.^46^ Autophagy is the homeostatic process by which cells are able to degrade old or damaged proteins and organelles, ensuring the continual presence of healthy ones in the cell.^47^ A decline in autophagy is generally associated with cell ageing. And geriatric satellite cells have a misregulation of autophagy, as observed by their inability to form new autophagosomes.^46^ However, restoring autophagy activity in satellite cells using rapamycin prevented them from entering senescence when aged to geriatric levels. Therefore, there is great interest in discovering the pathways that are involved in autophagy to further treat them and to better understand the cells response to certain environmental conditions.

### CARM1 Activates Autophagy Through AMPK

**Figure 3.**
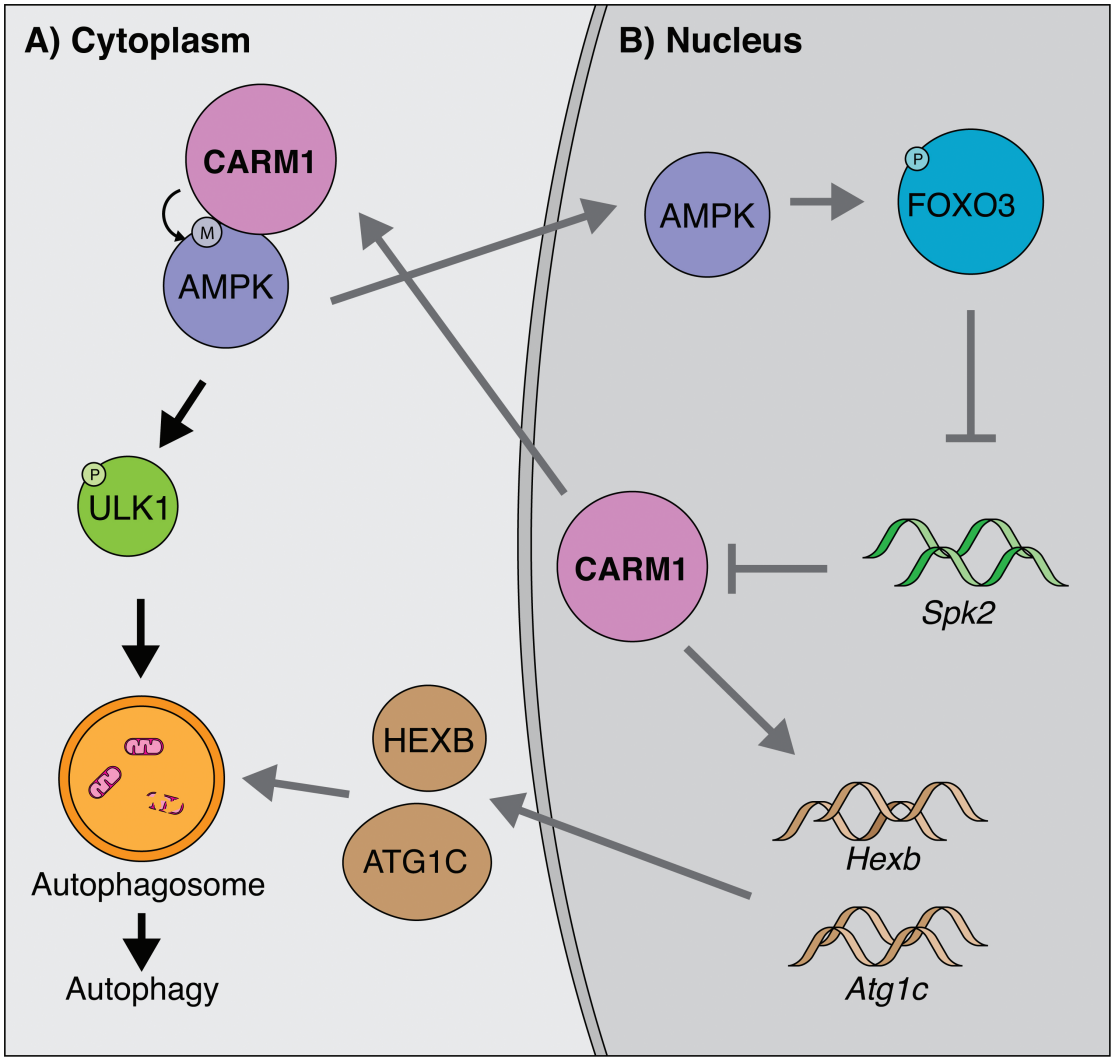
CARM1 function in autophagy. (**A**) Black arrows. In the cytoplasm, CARM1 methylates AMPK, which results in the phosphorylation of ULK1. This promotes the formation of autophagosomes leading to the degradation of aged organelles. (**B**) Gray arrows. In the nucleus, AMPK can phosphorylate FOXO3. This represses the expression of *Spk2*, leading to increased stability of CARM1. CARM1 can then positively regulate the expression of *Atg1c* and *Hexb* in the nucleus, which are involved in the formation of autophagosomes leading to autophagy. Moreover, increased CARM1 could feed-forward into autophagy although AMPK.

In recent years, CARM1’s role has expanded to include the regulation of autophagy. This occurs in a multifaceted way. For one, CARM1 can directly methylate AMPK, which is an important protein involved in the activation of autophagy in general and in skeletal muscle (Fig. 3A).^48,49^ Knockout of CARM1 abrogates this interaction and leads to a reduction in phosphorylation of AMPK targets, such as ULK1, an important protein involved in the early steps in the production of autophagosomes. Secondly, CARM1 has histone methyltransferase functions in the regulation of autophagy.^50^ During nutrient starvation of the cell, AMPK activation results in the phosphorylation of FOXO3 in the nucleus. This represses the expression of SKP2 which leads to an increase in CARM1 protein that methylates histone in a TFEB-dependant manner at autophagy and lysosome genes, such as *Atg1c* and *Hexb* (Fig. 3B). Consequently, the expression of these genes is increased.

Interestingly, there may exist a feedback loop between FOXO3 and CARM1. In the context of skeletal muscle wasting, CARM1 expression is positively correlated with worsening muscle conditions.^51^ Knockdown of CARM1 seems to attenuate some muscle wasting, which is perplexing given the important role CARM1 plays in muscle homeostasis. Of note, CARM1 interacts directly with FOXO3 and methylates it, which is important for its transcriptional activity in regulating its target genes such as *Antrogin-1* and *MuRF1*, 2 genes involved in atrophy. Moreover, CARM1 is still important for maintaining autophagy as seen by the reduction in LC3-II formation following CARM1 knockdown. Therefore, FOXO3 seems to have 2 types of interactions with CARM1. The first being FOXO3-mediated CARM1 expression, which is important for autophagy, and the second is CARM1-mediated methylation of FOXO3, which is involved in muscle atrophy. It is also likely that under pathological conditions such as muscle atrophy, the involvement of autophagy in addition to atrophy is responsible for exacerbating muscle wasting. Lastly, siRNA knockdown of *Carm1* in C2C12 myoblasts was associated with a reduction in the expression of *Ampk*, suggesting an additional mechanism by which CARM1 can regulate autophagy and feedback into its own expression.^19^

### Misregulated Autophagy Is involved in Duchenne Muscular Dystrophy

In addition to stimulating autophagy for the maintenance of muscle homeostasis in ageing, many have suggested that autophagy could be used as a therapeutic approach to treat Duchenne muscular dystrophy, another progressive disease affecting muscle through an impairment of regeneration in addition to sustained damage. Muscle from *mdx* mice and humans suffering from Duchenne have an impairment in autophagy, coupled with stimulation of AKT and mTOR pathways, which are also inhibitory to autophagy as evidenced by a reduction on autophagy-related gene expression of *Lc3* and *Atg12*.^52^ A low protein diet was able to reduce the activation of AKT and mTOR, increase the expression of LC3-II and rescue autophagy, leading to a reduction in symptoms of Duchenne such as inflammation and myofiber damage. AICAR (5-aminoimidazole-4-carboxamide-1-β-d-ribofuranoside) activation of the AMPK pathway has also been shown to restore autophagy in *mdx* mouse diaphragm.^53^ However, unlike long-term low protein diet, AICAR treatment does not seem to lead to muscle atrophy. In fact, AICAR has also been used to switch muscle fiber types to a more oxidative profile in an attempt to render them more resilient to damage induced by the lack of dystrophin.^54,55^ Finally, nanoparticles loaded with rapamycin is another autophagy-stimulating drug that has been used with beneficial outcomes in muscular dystrophy and ageing.^56^

It is possible that one of the consequences of CARM1 misregulation through its hyperphosphorylation and sequestration in Duchenne muscular dystrophy is an impairment in the autophagy pathways. As previously mentioned, CARM1 can mediate autophagy through the AMPK pathway, and this is already misregulated in the context of Duchenne muscular dystrophy. This represents an unexplored avenue for future research.

In summary, CARM1 is an important factor in the AMPK pathway leading to favorable outcomes in satellite cells and muscle. CARM1 activates AMPK by methylation, which in turn can activate the autophagy pathway to clear damaged organelles and prevent satellite cell senescence. Thus, restoration of CARM1 activity and the subsequent activation of autophagy may ameliorate dystrophic progression.

## Conclusion

CARM1 is a methyltransferase that functions as a coactivator in the regulation of gene expression. Notably, CARM1 plays an important regulatory role in the function of satellite cells by epigenetically regulating the function of PAX7 in specifying the transcriptional identity of the committed daughter cell following an asymmetric division. CARM1 is also important in the regulation of pre-mRNA splicing and autophagy and has important functions in embryonic development. In the context of disease, CARM1 has already been shown to be misregulated during satellite cell asymmetric divisions in DMD, with the potential for CARM1’s other functions to be misregulated. Moreover, various functions of CARM1 may be interacting together synergistically to modulate satellite cell function. For example, cytoplasmic CARM1 in the *Myf5* negative satellite cell may lead to a reduction in alternative splicing, as previously mentioned. On the other hand, that same cell may be more likely to promote AMPK-mediated autophagy occurring in the cytoplasm, ensuring the long-term maintenance of the more “stem” population of satellite cells. These unknowns leave many avenues for further exploration. However, limitations in our current knowledge mean that many roles discussed are purely theoretical. Therefore, exploring topics like autophagy and pre-mRNA splicing in quiescent and activated satellite cells is paramount to complete our knowledge of CARM1’s role during an asymmetric division.

## Data Availability

No new data were generated or analyzed in support of this research.

## References

[1] Liu L , Cheung TH, Charville GW, et al. Chromatin modifications as determinants of muscle stem cell quiescence and chronological aging. Cell Rep 2013;4(1):189-204. 23810552 10.1016/j.celrep.2013.05.043 PMC4103025

[2] Chen D , Ma H, Hong H, et al. Regulation of transcription by a protein methyltransferase. Science 1999;284(5423):2174-2177. 10381882 10.1126/science.284.5423.2174

[3] Cheng D , Cote J, Shaaban S, et al. The arginine methyltransferase CARM1 regulates the coupling of transcription and mRNA processing. Mol Cell. 2007;25(1):71-83. 17218272 10.1016/j.molcel.2006.11.019

[4] Xu W , Cho H, Kadam S, et al. A methylation-mediator complex in hormone signaling. Genes Dev. 2004;18(2):144-156. 14729568 10.1101/gad.1141704 PMC324421

[5] Lee YH , Coonrod SA, Kraus WL, et al. Regulation of coactivator complex assembly and function by protein arginine methylation and demethylimination. Proc Natl Acad Sci USA. 2005;102(10):3611-3616. 15731352 10.1073/pnas.0407159102 PMC553305

[6] Xu W , Chen H, Du K, et al. A transcriptional switch mediated by cofactor methylation. Science 2001;294(5551):2507-2511. 11701890 10.1126/science.1065961

[7] Wu J , Cui N, Wang R, et al. A role for CARM1-mediated histone H3 arginine methylation in protecting histone acetylation by releasing corepressors from chromatin. PLoS One. 2012;7(6):e34692. 22723830 10.1371/journal.pone.0034692 PMC3377634

[8] Mauro A. Satellite cell of skeletal muscle fibers. J Biophys Biochem Cytol 1961;9:493-495. 13768451 10.1083/jcb.9.2.493 PMC2225012

[9] Seale P , Sabourin LA, Girgis-Gabardo A, et al. Pax7 is required for the specification of myogenic satellite cells. Cell 2000;102(6):777-786. [In English]. 11030621 10.1016/s0092-8674(00)00066-0

[10] Kuang S , Charge SB, Seale P, et al. Distinct roles for Pax7 and Pax3 in adult regenerative myogenesis. J Cell Biol. 2006;172(1):103-113. [in English]. 16391000 10.1083/jcb.200508001 PMC2063538

[11] Relaix F , Montarras D, Zaffran S, et al. Pax3 and Pax7 have distinct and overlapping functions in adult muscle progenitor cells [in eng]. J Cell Biol. 2006;172(1):91-102. 16380438 10.1083/jcb.200508044 PMC2063537

[12] Schultz E , Gibson MC, Champion T. Satellite cells are mitotically quiescent in mature mouse muscle: an EM and radioautographic study. J Exp Zool. 1978;206(3):451-456. 712350 10.1002/jez.1402060314

[13] Bentzinger CF , Wang YX, Rudnicki MA. Building muscle: molecular regulation of myogenesis. Cold Spring Harb Perspect Biol 2012;4(2):a008342-a008342. 22300977 10.1101/cshperspect.a008342 PMC3281568

[14] Pang L , Tian H, Chang N, et al. Loss of CARM1 is linked to reduced HuR function in replicative senescence. BMC Mol Biol. 2013;14(1):15. 23837869 10.1186/1471-2199-14-15 PMC3718661

[15] Chen SL , Loffler KA, Chen D, et al. The coactivator-associated arginine methyltransferase is necessary for muscle differentiation: CARM1 coactivates myocyte enhancer factor-2. J Biol Chem. 2002;277(6):4324-4333. 11713257 10.1074/jbc.M109835200

[16] Gao X , Pan WS, Dai H, et al. CARM1 activates myogenin gene via PCAF in the early differentiation of TPA-induced rhabdomyosarcoma-derived cells. J Cell Biochem. 2010;110(1):162-170. 20213728 10.1002/jcb.22522

[17] Dacwag CS , Ohkawa Y, Pal S, et al. The protein arginine methyltransferase Prmt5 is required for myogenesis because it facilitates ATP-dependent chromatin remodeling. Mol Cell Biol. 2007;27(1):384-394. 17043109 10.1128/MCB.01528-06 PMC1800640

[18] Dacwag CS , Bedford MT, Sif S, et al. Distinct protein arginine methyltransferases promote ATP-dependent chromatin remodeling function at different stages of skeletal muscle differentiation. Mol Cell Biol. 2009;29(7):1909-1921. 19188441 10.1128/MCB.00742-08 PMC2655603

[19] Wang SC , Dowhan DH, Eriksson NA, et al. CARM1/PRMT4 is necessary for the glycogen gene expression programme in skeletal muscle cells. Biochem J. 2012;444(2):323-331. 22428544 10.1042/BJ20112033

[20] Kuang S , Kuroda K, Le Grand F, et al. Asymmetric self-renewal and commitment of satellite stem cells in muscle. Cell 2007;129(5):999-1010. 17540178 10.1016/j.cell.2007.03.044 PMC2718740

[21] Dumont NA , Wang YX, von Maltzahn J, et al. Dystrophin expression in muscle stem cells regulates their polarity and asymmetric division. Nat Med. 2015;21(12):1455-1463. 26569381 10.1038/nm.3990 PMC4839960

[22] Chang NC , Sincennes MC, Chevalier FP, et al. The dystrophin glycoprotein complex regulates the epigenetic activation of muscle stem cell commitment. Cell Stem Cell 2018;22(5):755-768 e756. 29681515 10.1016/j.stem.2018.03.022 PMC5935555

[23] Kawabe Y , Wang YX, McKinnell IW, et al. Carm1 regulates Pax7 transcriptional activity through MLL1/2 recruitment during asymmetric satellite stem cell divisions. Cell Stem Cell 2012;11(3):333-345. 22863532 10.1016/j.stem.2012.07.001 PMC3438319

[24] McKinnell IW , Ishibashi J, Le Grand F, et al. Pax7 activates myogenic genes by recruitment of a histone methyltransferase complex. Nat Cell Biol. 2008;10(1):77-84. 18066051 10.1038/ncb1671 PMC2739814

[25] Conboy IM , Rando TA. The regulation of Notch signaling controls satellite cell activation and cell fate determination in postnatal myogenesis. Dev Cell. 2002;3(3):397-409. 12361602 10.1016/s1534-5807(02)00254-x

[26] Bjornson CR , Cheung TH, Liu L, et al. Notch signaling is necessary to maintain quiescence in adult muscle stem cells. Stem Cells. 2012;30(2):232-242. 22045613 10.1002/stem.773 PMC3384696

[27] Mourikis P , Sambasivan R, Castel D, et al. A critical requirement for notch signaling in maintenance of the quiescent skeletal muscle stem cell state. Stem Cells. 2012;30(2):243-252. 22069237 10.1002/stem.775

[28] Hein K , Mittler G, Cizelsky W, et al. Site-specific methylation of Notch1 controls the amplitude and duration of the Notch1 response. Sci Signal 2015;8(369):ra30. 25805888 10.1126/scisignal.2005892

[29] Kottlors M , Kirschner J. Elevated satellite cell number in Duchenne muscular dystrophy. Cell Tissue Res. 2010;340(3):541-548. 20467789 10.1007/s00441-010-0976-6

[30] Wu Q , Bruce AW, Jedrusik A, et al. CARM1 is required in embryonic stem cells to maintain pluripotency and resist differentiation. Stem Cells. 2009;27(11):2637-2645. 19544422 10.1002/stem.131 PMC4135545

[31] Rescan PY , Montfort J, Fautrel A, et al. Gene expression profiling of the hyperplastic growth zones of the late trout embryo myotome using laser capture microdissection and microarray analysis. BMC Genomics. 2013;14(1):173. 23497127 10.1186/1471-2164-14-173 PMC3608082

[32] Batut J , Duboe C, Vandel L. The methyltransferases PRMT4/CARM1 and PRMT5 control differentially myogenesis in zebrafish. PLoS One. 2011;6(10):e25427. 22016767 10.1371/journal.pone.0025427 PMC3189919

[33] Han J , Xiong J, Wang D, et al. Pre-mRNA splicing: where and when in the nucleus. Trends Cell Biol. 2011;21(6):336-343. 21514162 10.1016/j.tcb.2011.03.003 PMC6553873

[34] Fujiwara T , Mori Y, Chu DL, et al. CARM1 regulates proliferation of PC12 cells by methylating HuD. Mol Cell Biol. 2006;26(6):2273-2285. 16508003 10.1128/MCB.26.6.2273-2285.2006 PMC1430293

[35] Ohkura N , Takahashi M, Yaguchi H, et al. Coactivator-associated arginine methyltransferase 1, CARM1, affects pre-mRNA splicing in an isoform-specific manner. J Biol Chem. 2005;280(32):28927-28935. 15944154 10.1074/jbc.M502173200

[36] Kuhn P , Chumanov R, Wang Y, et al. Automethylation of CARM1 allows coupling of transcription and mRNA splicing. Nucleic Acids Res. 2011;39(7):2717-2726. 21138967 10.1093/nar/gkq1246 PMC3074151

[37] Wang L , Charoensuksai P, Watson NJ, et al. CARM1 automethylation is controlled at the level of alternative splicing. Nucleic Acids Res. 2013;41(14):6870-6880. 23723242 10.1093/nar/gkt415 PMC3737532

[38] Chen K , Dai X, Wu J. Alternative splicing: an important mechanism in stem cell biology. World J Stem Cells 2015;7(1):1-10. 25621101 10.4252/wjsc.v7.i1.1 PMC4300919

[39] Chen L , Kostadima M, Martens JHA, et al. Transcriptional diversity during lineage commitment of human blood progenitors. Science 2014;345(6204):1251033. 25258084 10.1126/science.1251033 PMC4254742

[40] Li Y , Wang D, Wang H, et al. A splicing factor switch controls hematopoietic lineage specification of pluripotent stem cells. EMBO Rep. 2021;22(1):e50535. 33319461 10.15252/embr.202050535 PMC7788460

[41] Su CH , D D, Tarn WY. Alternative splicing in neurogenesis and brain development. Front Mol Biosci 2018;5:12. [review] [in English] 29484299 10.3389/fmolb.2018.00012 PMC5816070

[42] Sanchez G , Bondy-Chorney E, Laframboise J, et al. A novel role for CARM1 in promoting nonsense-mediated mRNA decay: potential implications for spinal muscular atrophy. Nucleic Acids Res. 2016;44(6):2661-2676. 26656492 10.1093/nar/gkv1334 PMC4824080

[43] Renault V , Thornell LE, Eriksson PO, et al. Regenerative potential of human skeletal muscle during aging. Aging Cell. 2002;1(2):132-139. 12882343 10.1046/j.1474-9728.2002.00017.x

[44] Jang YC , Sinha M, Cerletti M, et al. Skeletal muscle stem cells: effects of aging and metabolism on muscle regenerative function. Cold Spring Harb Symp Quant Biol. 2011;76(0):101-111. 21960527 10.1101/sqb.2011.76.010652

[45] Sousa-Victor P , Gutarra S, Garcia-Prat L, et al. Geriatric muscle stem cells switch reversible quiescence into senescence. Nature 2014;506(7488):316-321. 24522534 10.1038/nature13013

[46] Garcia-Prat L , Martinez-Vicente M, Perdiguero E, et al. Autophagy maintains stemness by preventing senescence. Nature 2016;529(7584):37-42. 26738589 10.1038/nature16187

[47] Cuervo AM , Bergamini E, Brunk UT, et al. Autophagy and aging: the importance of maintaining “clean” cells. Autophagy 2005;1(3):131-140. 16874025 10.4161/auto.1.3.2017

[48] Kjobsted R , Hingst JR, Fentz J, et al. AMPK in skeletal muscle function and metabolism. FASEB J. 2018;32(4):1741-1777. 29242278 10.1096/fj.201700442R PMC5945561

[49] Stouth DW , vanLieshout TL, Ng SY, et al. CARM1 regulates AMPK signaling in skeletal muscle. iScience 2020;23(11):101755. 33241200 10.1016/j.isci.2020.101755 PMC7672286

[50] Shin HJ , Kim H, Oh S, et al. AMPK-SKP2-CARM1 signalling cascade in transcriptional regulation of autophagy. Nature 2016;534(7608):553-557. 27309807 10.1038/nature18014 PMC5568428

[51] Liu Y , Li J, Shang Y, et al. CARM1 contributes to skeletal muscle wasting by mediating FoxO3 activity and promoting myofiber autophagy. Exp Cell Res. 2019;374(1):198-209. 30500392 10.1016/j.yexcr.2018.11.024

[52] De Palma C , Morisi F, Cheli S, et al. Autophagy as a new therapeutic target in Duchenne muscular dystrophy. Cell Death Dis. 2012;3(11):e418. 23152054 10.1038/cddis.2012.159 PMC3542595

[53] Pauly M , Daussin F, Burelle Y, et al. AMPK activation stimulates autophagy and ameliorates muscular dystrophy in the mdx mouse diaphragm. Am J Pathol. 2012;181(2):583-592. 22683340 10.1016/j.ajpath.2012.04.004

[54] Webster C , Silberstein L, Hays AP, et al. Fast muscle fibers are preferentially affected in Duchenne muscular dystrophy. Cell 1988;52(4):503-513. 3342447 10.1016/0092-8674(88)90463-1

[55] Ljubicic V , Miura P, Burt M, et al. Chronic AMPK activation evokes the slow, oxidative myogenic program and triggers beneficial adaptations in mdx mouse skeletal muscle. Hum Mol Genet. 2011;20(17):3478-3493. 21659335 10.1093/hmg/ddr265

[56] Bibee KP , Cheng YJ, Ching JK, et al. Rapamycin nanoparticles target defective autophagy in muscular dystrophy to enhance both strength and cardiac function. FASEB J. 2014;28(5):2047-2061. 24500923 10.1096/fj.13-237388 PMC3986846

